# Modulating immunometabolism of tumor specific CD8 T cells to enhance T cell based therapy for cancer

**DOI:** 10.1186/2051-1426-2-S3-O2

**Published:** 2014-11-06

**Authors:** Madhusudhanan Sukumar, Jie Liu, Gautam Mehta, Luca Gattinoni, Toren Finkel, Nicholas Restifo

**Affiliations:** 1 National Cancer Institute, Bethesda, MD, USA; 2 National Institutes of Health, Bethesda, MD, USA; 3 National Cancer Institute, National Institutes of Health, Bethesea, MD, USA

## 

The ability of tumor-reactive CD8^+ ^T cells to eradicate tumors following adoptive transfer of autologous tumor-infiltrating lymphocytes correlates with their capacity to proliferate and persist for long periods of time. These qualities are found predominantly in naive and less differentiated memory cells including memory stem cells (T_SCM_) and central memory cells (T_CM_), but the metabolic control of differentiation remains unknown. Identification of distinct functional T cell subsets has historically relied on cell surface markers. Here, we show that combining such immunophenotyping approaches with an assessment of mitochondrial activity enables significant enrichment of stem cell-like T cell populations with enhanced *in vivo *biological activity. Upon antigenic stimulation, naïve CD8^+ ^T cells divert their bioenergetic metabolism from oxidative phosphorylation to aerobic glycolysis. Enforcing glycolytic metabolism by overexpressing the glycolytic enzyme phosphoglycerate mutase-1 (PGAM-1) severely impaired the ability of CD8^+ ^T cells to form long-term memory. Conversely, activation of CD8^+ ^T cells in the presence of an inhibitor of glycolysis, 2-deoxyglucose, enhanced the generation of memory cells and antitumor functionality. Furthermore, using TMRM, a fluorescent dye that measures mitochondrial potential in T cells, we found that mitochondrial membrane potential (MP) and reactive oxygen species in T cells critically control T cell longevity. Cells with lower membrane potential ('low MP') had a molecular profile characteristic of stem-cell memory precursors and displayed an enhanced ability to enter the memory pool as compared to cells displaying higher mitochondrial potential ('high MP') characteristic of short-lived effectors. Global metabolomic and functional studies revealed that 'low MP' cells exhibited increased levels of intracellular free fatty acid metabolites, increased expression of CPT-1a, a rate limiting enzyme involved in fatty acid oxidation and increased mitochondrial spare respiratory capacity, a metabolic property characteristic of long lived memory T cells. In comparison, 'high MP' T cells displayed enhanced lactate production. Most importantly, we observed a 100 fold increase in the frequency of secondary memory CD8^+ ^T cells 300 days after adoptive transfer of 'low MP' as compared to 'high MP' T cells. In tumor-bearing mice, 'low MP' cells exhibited increased cytokine functionality and resulted in the regression of large, vascularized tumors. Our findings therefore establish low ΔΨm as a hallmark of stem cell-like behavior and provide a simple, general and robust enrichment strategy based on intrinsic cellular metabolism that could have widespread applications in both regenerative medicine and to improve T cell based immunotherapy for cancer

**Figure 1:**
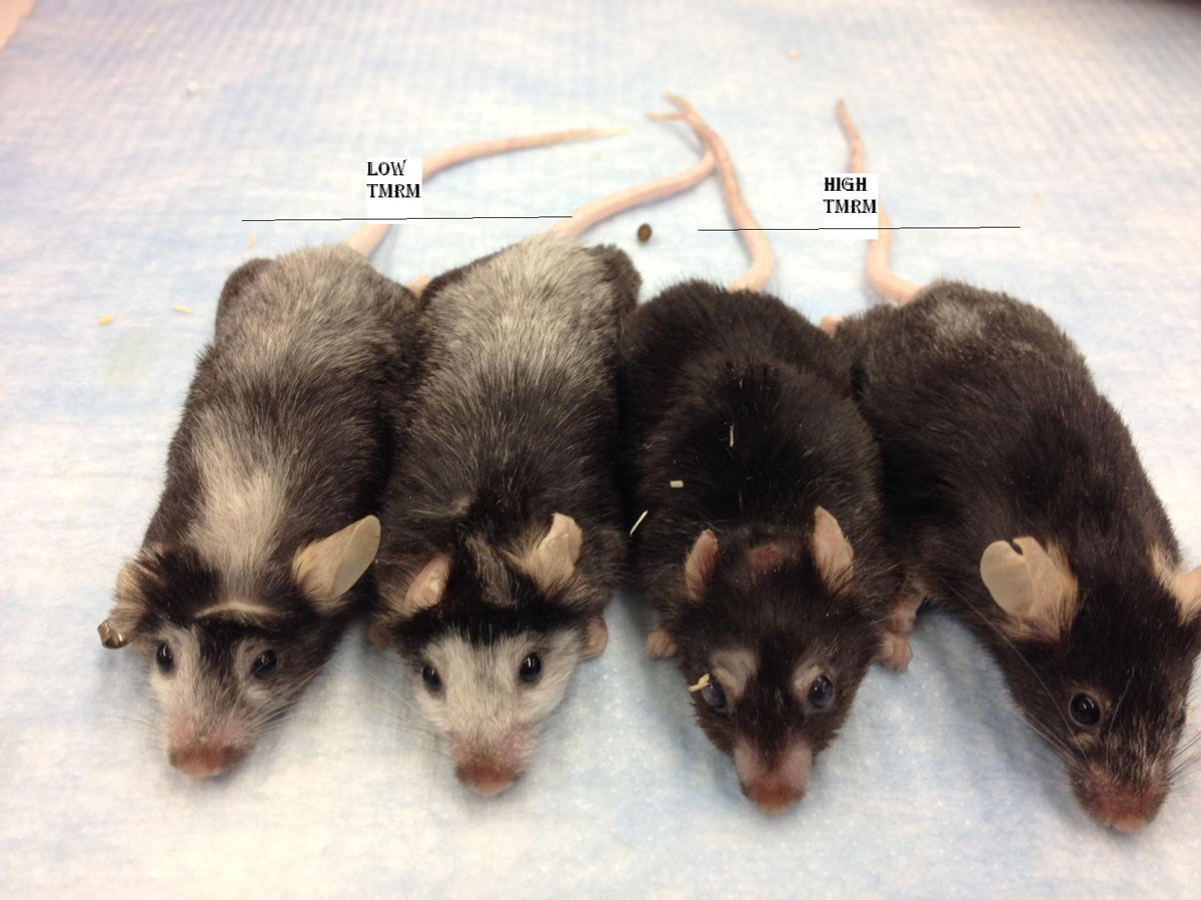
Degree of autoimmune-induced vitiligo 300 days after adoptive transfer of low TMRM (left) and high-TMRM (right) CD8+ T cells

